# Endotrophin as a risk marker of mortality and kidney complications in a type 1 diabetes cohort

**DOI:** 10.3389/fmolb.2023.1229579

**Published:** 2023-09-01

**Authors:** Alexandra Louise Møller, Ninna Hahn Tougaard, Daniel Guldager Kring Rasmussen, Federica Genovese, Pernille Falberg Rønn, Tine Willum Hansen, Morten Asser Karsdal, Peter Rossing

**Affiliations:** ^1^ Nordic Bioscience, Herlev, Denmark; ^2^ Department of Biomedical Sciences, Faculty of Health and Medical Sciences, University of Copenhagen, Copenhagen, Denmark; ^3^ Steno Diabetes Center Copenhagen, Herlev, Denmark; ^4^ Department of Clinical Medicine, Faculty of Health and Medical Sciences, University of Copenhagen, Copenhagen, Denmark

**Keywords:** endotrophin, fibrosis, biomarker, extracellular matrix, collagen, diabetes complications

## Abstract

Hyperglycemia triggers pathological pathways leading to fibrosis, where extracellular matrix (ECM) components are accumulated. We investigated the potential of endotrophin, a pro-fibrotic molecule generated during collagen type VI formation, as a risk marker for complications to type 1 diabetes. Endotrophin was measured in serum and urine from 1,468 persons with type 1 diabetes. Outcomes included a composite kidney endpoint, first major adverse cardiovascular event (MACE), all-cause mortality, progression of albuminuria, incident heart failure, and sight-threatening diabetic eye disease. Cox proportional hazards models adjusted for conventional risk factors were applied. A doubling of serum endotrophin was independently associated with the kidney endpoint (*n* = 30/1,462; hazard ratio 3.39 [95% CI: 1.98–5.82]), all-cause mortality (*n* = 93/1,468; 1.44 [1.03–2.0]), and progression of albuminuria (*n* = 80/1,359; 1.82 [1.32–2.52]), but not with first MACE, heart failure, or sight-threatening diabetic eye disease after adjustment. Urinary endotrophin was not associated with any outcome after adjustment. Serum endotrophin was a risk marker for mortality and kidney complications in type 1 diabetes. Biomarkers of ECM remodeling, such as serum endotrophin, may identify persons with active pro-fibrotic processes at risk for complications in diabetes and where antifibrotic agents may reduce this risk.

## Introduction

Persons with diabetes are at increased risk of complications related to the micro- and macrovascular circulation. The primary cause of premature mortality in persons with diabetes is cardiovascular disease (Baena-Díez et al., 2016), and chronic kidney disease (CKD) increase the overall risk of cardiovascular complications (Groop et al., 2009). Early intervention targeting several risk factors has been implemented, but new tools to predict complications before clinical manifestations are needed to improve risk stratification and personalize preventive treatment.

Hyperglycemia drives oxidative stress, inflammation, and tissue injury, eventually leading to fibrosis, characterized by an abnormal shift in the turnover of components of the extracellular matrix (ECM). However, differences in glycemic control do not fully explain the variation in the incidence and severity of complications. Despite the underlying etiology, kidney disease development is assumed to be driven by kidney fibrosis. As formation and degradation of ECM components are linked with the development of fibrosis (Bülow and Boor, 2019), assessment of markers of ECM turnover may identify persons with active pro-fibrotic processes at higher risk for complications related to diabetes. During fibrosis progression, peptides reflecting ECM remodeling, such as collagens, are released (Genovese et al., 2014). Collagens are an essential part of the fibrotic tissue, acting as both a scaffold for cells and an interaction partner for several proteins (Gelse et al., 2003; Liu, 2011; Genovese et al., 2014).

Interestingly, specific proteolysis-derived fragments of collagens can have vital signaling functions (Karsdal et al., 2015). The bioactive fragment, endotrophin, released during collagen type VI synthesis when the C-terminal pro-peptide of the α3 chain is cleaved off from the mature molecule (Aigner et al., 2002), is pro-inflammatory and pro-fibrotic (Park and Scherer, 2012; Karousou et al., 2014; Zhao et al., 2016; Funcke and Scherer, 2019). Endotrophin is expressed by cells from the mesenchymal stem cells, including adipocytes and fibroblasts (Juhl et al., 2020; Møller, 2020; Rønnow et al., 2020), suggesting it to be a central ECM fragment associated with chronic fibro-inflammatory diseases.

In this large and unselected study cohort, including persons with type 1 diabetes, we aimed to validate the previous findings of endotrophin in type 1 diabetes (Pilemann-Lyberg et al., 2019) and investigate whether higher levels of serum and urinary endotrophin were associated with risk of progression of complications to type 1 diabetes.

## Materials and methods

### Research design and methods

The study is based on data and biobank material from the StenoDot cohort recruited from the outpatient clinic at Steno Diabetes Center Copenhagen, Denmark from 2012 to 2016. The present study complied with the Declaration of Helsinki and was approved by The Regional Ethics Committee in The Capital Region of Denmark (H-19042436). Detailed information about the research design and International Statistical Classification of Diseases (ICD)- and procedural codes for baseline and follow-up measures has previously been published (Tougaard et al., 2022). In short, the present study cohort included 1,468 individuals with type 1 diabetes, defined as an E10 diagnosis (ICD-10); or an E13 or E14 diagnosis, treated solely with insulin. Urinary albumin excretion rate (mg/24 h) and urine albumin-creatinine ratio (mg/g) were considered comparable measures and were pooled as a composite variable of urinary albumin excretion (UAE) (mg/g).

Demographic and clinical data were extracted from patient records, including retinal photo gradings and routine laboratory measurements. Information on hospital admissions, emigration, and deaths was obtained from national registers. Primary endpoints were 1) a composite kidney endpoint defined as estimated glomerular filtration rate (eGFR) decline of ≥40% confirmed after minimum 1 month or unconfirmed if the measurement was the last before end-of-follow-up, development of CKD stage 5, chronic dialysis, kidney transplantation, or kidney failure as cause of death, 2) first major adverse cardiovascular event (MACE) including cardiovascular death, non-fatal acute myocardial infarction, coronary intervention, and non-fatal stroke, and 3) all-cause mortality. Secondary endpoints were 1) progression in albuminuria stage based on minimum one measurement, 2) incident heart failure, and 3) incident sight-threatening diabetic eye disease, including proliferative retinopathy and maculopathy based on retinal photos or procedural codes. Median follow-up was 6.4 years for the composite kidney endpoint, 6.3 years for MACE, 5.3 years for all-cause mortality, 6.3 years for albuminuria progression, 6.4 years for incident heart failure, and 3.1 years for sight-threatening diabetic eye disease. ICD-8, ICD-10, and procedural codes are provided in Supplementary Table S1.

Levels of endotrophin were measured in serum and urine at baseline using the PRO-C6 enzyme-linked immunosorbent assay (ELISA) (Nordic Bioscience, Herlev, Denmark) (Sun et al., 2015). The ELISA was carried out as previously described (Sun et al., 2015). Urinary endotrophin levels were normalized to urinary creatinine levels. Urinary creatinine was measured using the ADVIA 1800 Clinical Chemistry System.

### Statistical analyses

Baseline clinical characteristics were stratified by tertiles of serum and urinary endotrophin levels, respectively. All continuous clinical variables, except UAE, were normally distributed, and levels were presented as mean ± standard deviation (SD), whereas UAE levels were presented as median with median interquartile range (IQR). Categorical variables were presented as total numbers with corresponding percentages. Differences among tertiles were assessed with one-way ANOVA for normally distributed variables, the Kruskal–Wallis test for non-normally distributed variables, and the χ^2^ test for categorical variables. The correlation between serum endotrophin and urinary endotrophin was tested by Spearman’s rank correlation coefficient.

In the longitudinal analyses, participants were followed until an event or censoring due to emigration, death, or end of follow-up. The association between a doubling of serum endotrophin and urinary endotrophin and incidence of the specified endpoints during follow-up was investigated by Cox proportional hazards regression analysis, both unadjusted and adjusted for the conventional risk factors sex, baseline age, body mass index (BMI), low-density lipoprotein (LDL)-cholesterol, current smoking, hemoglobin A1c (HbA1c), systolic blood pressure, eGFR, and UAE (except for analyses of albuminuria progression). For each outcome, participants previously diagnosed with the outcome were excluded. The distribution of serum endotrophin, urinary endotrophin, and UAE was skewed, and these variables were log2-transformed before analyses. All two-tailed *p* < 0.05 were considered significant. Statistical analyses were performed using R (4.1.0).

## Results

The cohort included 1,468 persons with type 1 diabetes. Serum samples were available for 1,446 persons (99%) of the total cohort and consisted of 712 (49%) females, mean age was 51 ± SD of 16 years, diabetes duration 26 ± 15 years, HbA1c 62 ± 12 mmol/mol, eGFR 94 ± 23 mL/min/1.73 m, and IQR of the last UAE was 5.5 (3.5–11.5) mg/g. The cohort with available serum samples consisted of 1,080 (75%) persons with normoalbuminuria, 260 (18%) with microalbuminuria, and 106 (7%) with macroalbuminuria. Urine samples were available for 1,251 persons (85%) of the total cohort.

Baseline characteristics stratified by serum endotrophin are shown in Table 1. Higher levels of serum endotrophin were associated with higher age, diabetes duration, BMI, UAE, systolic blood pressure; lower eGFR; a higher proportion were prescribed anti-hypertensive, renin-angiotensin-aldosterone system (RAAS) blockade, and lipid-lowering treatment; and a higher proportion with a history of MACE and sight-threatening diabetic eye disease (Table 1). Urinary endotrophin levels stratified into tertiles were not associated with markers of disease severity (Supplementary Table S2).

**TABLE 1:** Clinical characteristics stratified by serum endotrophin tertiles.

Characteristic	T1 (*n* = 482)	T2 (*n* = 482)	T3 (*n* = 482)	*P*
Serum endotrophin (ng/mL)	6 (5–6)	8 (8–9)	13 (11–17)	
Age (years)	48 ± 15	50 ± 17	53 ± 16	**< 0.001**
Female sex (%)	215 (45)	240 (50)	249 (52)	0.076
BMI (kg/m^2^)	25 ± 3.7	26 ± 4.1	26 ± 4.4	**0.004**
Systolic blood pressure (mmHg)	128 ± 14.7	129 ± 15.6	131 ± 17.3	**0.029**
Diastolic blood pressure (mmHg)	78 ± 7.9	76 ± 9.1	76 ± 9.4	**< 0.001**
Diabetes duration (years)	22 ± 14	25 ± 14	30 ± 17	**< 0.001**
HbA1c (mmol/mol)	62.0 ± 12.9	61.8 ± 11.7	61.7 ± 12.0	0.928
UAE (mg/g)	5.5 (3.5–9.5)	4.5 (2.5–9.5)	6.5 (3.5–25.5)	**< 0.001**
eGFR (ml/min/1.73 m^2^)	101 ± 17	96 ± 19	84 ± 28	**< 0.001**
LDL-cholesterol (mmol/L)	2.5 ± 0.8	2.4 ± 0.7	2.4 ± 0.8	0.979
Current smoker (%)	122 (26)	77 (16)	64 (13)	**< 0.001**
Treatment				
Insulin (%)	476 (99)	474 (98)	473 (98)	0.734
Anti-hypertensives (%)	187 (39)	217 (45)	273 (57)	**< 0.001**
RAAS blockade (%)	162 (34)	200 (42)	238 (49)	**< 0.001**
Lipid-lowering medication (%)	199 (41)	224 (47)	262 (54)	**< 0.001**
Disease history				
MACE (%)	27 (6)	43 (9)	81 (17)	**< 0.001**
Sight-threatening diabetic eye disease (%)	66 (14)	78 (16)	150 (31)	**< 0.001**

Data are median (IQR), n (%), or mean ± SD., serum endotrophin levels are rounded to whole numbers due to sensitive personal data.

Significant differences are indicated in bold.

There was no correlation between serum and urinary endotrophin (Spearman *r* = 0.011, *p* = 0.70). As urinary endotrophin was corrected for urinary creatinine, we also analyzed serum endotrophin and urinary endotrophin not normalized for creatinine. There was no correlation between serum endotrophin and unadjusted urinary endotrophin (Spearman *r* = 0.044, *p* = 0.12).

**FIGURE 1:**
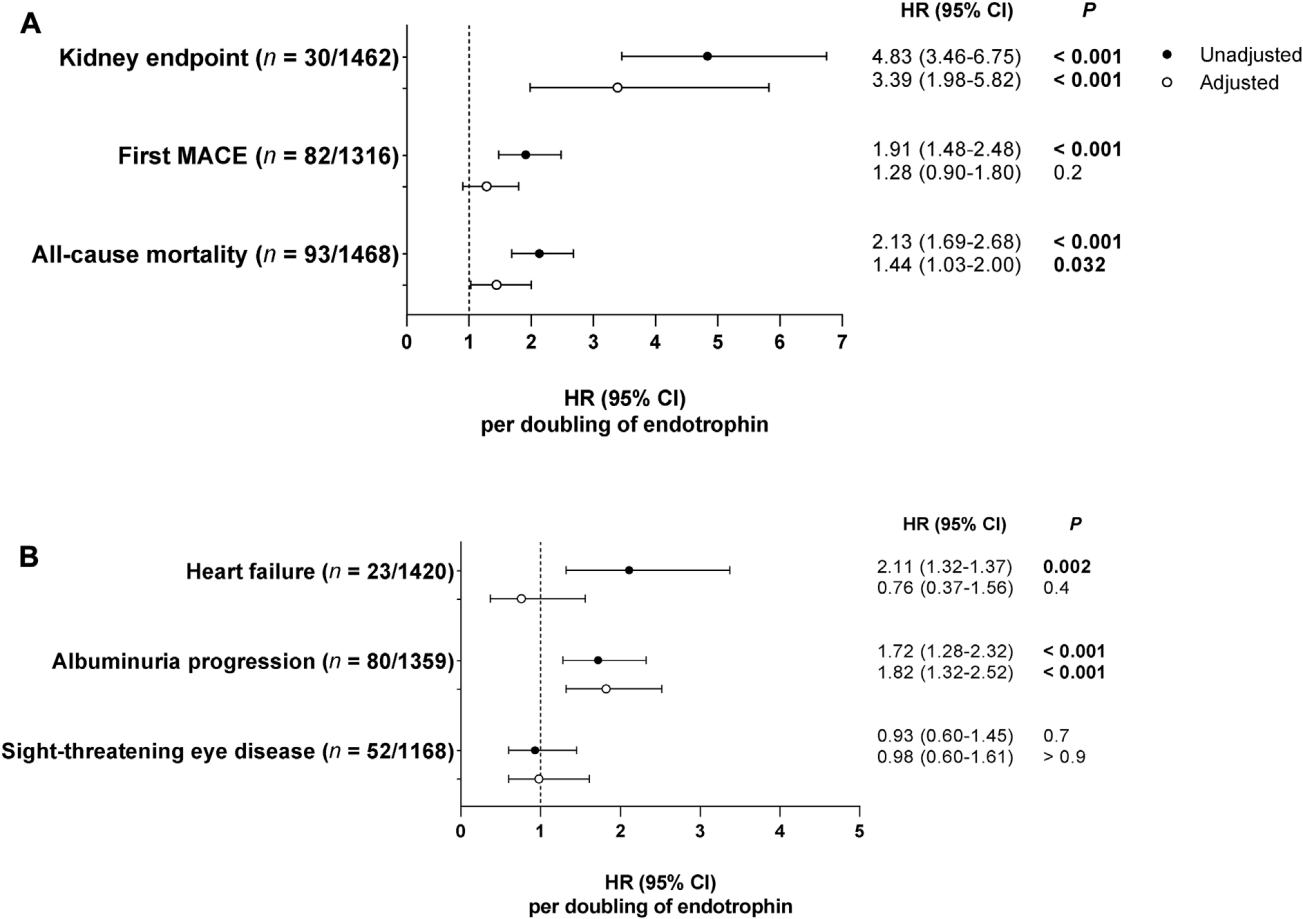
Associations between serum endotrophin and incidence of complications estimated by Cox proportional-hazards models. Hazard ratios (HRs) with 95% confidence intervals (CIs) are listed per doubling of serum endotrophin for the primary **(A)** and secondary **(B)** endpoints. HRs are reported as unadjusted and adjusted for the conventional risk factors sex, baseline age, BMI, LDL-cholesterol, current smoking, HbA1c, systolic blood pressure, eGFR, and UAE (except for analyses on albuminuria progression). For each outcome, participants previously diagnosed with the outcome were excluded.

The association between serum endotrophin and incidence of complications estimated by unadjusted and adjusted Cox proportional-hazards models is shown in Figure 1. Higher levels of serum endotrophin were significantly associated with the kidney endpoint and all-cause mortality in both unadjusted and adjusted analyses (Figure 1A). When adjusting for conventional risk factors, the hazard ratio (HR) per doubling of serum endotrophin was 3.39 [95% CI: 1.98–5.82] for the kidney endpoint (*n* = 30/1,462), 1.28 [0.90–1.80] for first MACE (*n* = 82/1,316), and 1.44 [1.03–2.0] for all-cause mortality (*n* = 93/1,468) (Figure 1A). For the secondary endpoints, there was an association between a doubling of serum endotrophin and progression of albuminuria (*n* = 80/1,359) with a HR of 1.82 [1.32–2.52] but not with incident heart failure (*n* = 23/1,420) or sight-threatening diabetic eye-disease (*n* = 52/1,168) after adjustment (Figure 1B). No significant association between urinary endotrophin and the specified endpoints was observed after adjustment (data not shown).

## Discussion

In a cohort of 1,468 persons with type 1 diabetes, we demonstrate that higher serum levels of a fragment reflecting collagen type VI formation and endotrophin are associated with a higher hazard of death and kidney complications. Even when adjusted for the conventional risk factors, sex, age, BMI, LDL-cholesterol, current smoking, HbA1c, systolic blood pressure, eGFR, and UAE (except for analyses on albuminuria progression), serum endotrophin remained significantly associated with all-cause mortality, the kidney endpoint, and progression of albuminuria. These findings align with previous results for circulating endotrophin (Rasmussen et al., 2018; Frimodt-Møller et al., 2019; Pilemann-Lyberg et al., 2019; Rasmussen et al., 2022; Tougaard et al., 2022), highlighting the evidence for endotrophin as a relevant risk marker for progression of CKD and the development or progression of complications in diabetes.

We validated the previous findings of serum endotrophin in type 1 diabetes, where higher levels of serum endotrophin were independently associated with a higher risk of mortality and development of end-stage kidney disease (Pilemann-Lyberg et al., 2019). Of note, the participants in the present study cohort had a lower baseline age, diabetes duration, UAE, and higher eGFR compared to the participants included in the previous study (Pilemann-Lyberg et al., 2019).

We have previously shown that serum endotrophin was associated with all-cause mortality and kidney and cardiovascular complications in persons with type 2 diabetes (Tougaard et al., 2022) and persons with type 2 diabetes and microalbuminuria (Rasmussen et al., 2018). Moreover, in the CANVAS trial, plasma endotrophin was an independent risk marker for incident heart failure, cardiovascular disease, the composite kidney endpoints, and all-cause mortality (Rasmussen et al., 2022). Taken together, the robust results of circulating endotrophin across diabetes cohorts are promising for its application as a risk marker for complications.

In the RIISC study, a prospective, observational cohort of persons with high-risk CKD, serum endotrophin was independently associated with mortality in CKD (Fenton et al., 2017), suggesting that collagen type VI formation and endotrophin is mechanistically involved in the increased mortality risk associated with CKD. Moreover, in the PERF study, a prospective, observational cohort of elderly women without diabetes, serum endotrophin was associated with chronic multimorbidity and mortality independent of age and BMI (Staunstrup et al., 2021).

Importantly, previous studies have shown that collagen type VI is accumulated in the kidneys of persons with kidney disease (Nerlich et al., 1994; Vleming et al., 1995; Mason and Wahab, 2003), and colocalization of endotrophin with collagen type VI in the fibrotic kidney has been confirmed (Rasmussen et al., 2017).

To our knowledge, human intervention studies targeting endotrophin have yet to be conducted. Still, neutralizing the pro-fibrotic endotrophin with antibodies has been suggested to slow the imbalanced ECM remodeling in fibrogenesis (Williams et al., 2021). Interestingly, recent data from a podocyte ablation model showed that endotrophin neutralization through targeted antibody treatment protects from kidney fibrosis (An et al., 2023), suggesting that neutralizing endotrophin is a promising therapy for intervening with kidney fibrosis in CKD.

Results from the NEFIGAN and AWARD-7 trials showed that budesonide and dulaglutide treatment, respectively, reduced levels of circulating endotrophin (Maixnerova et al., 2021; Tuttle et al., 2023), indicating that budesonide and dulaglutide may reduce fibrosis by diminishing collagen type VI formation and levels of endotrophin. The nonsteroidal mineralocorticoid receptor antagonist, finerenone, reducing kidney and cardiovascular fibrosis in experimental studies (Kolkhof et al., 2014; Grune et al., 2018), has recently been shown to reduce progression of kidney and cardiovascular complications in type 2 diabetes (Agarwal et al., 2022). Finerenone has not yet been investigated in type 1 diabetes; however, short-term studies with spironolactone in persons with type 1 diabetes and kidney disease demonstrated a reduction in albuminuria (Schjoedt et al., 2005). Thus, serum endotrophin may be used to select persons who could benefit from preventive treatment with finerenone.

The strength of this study is that endotrophin was measured in a large, unselected, and well-characterized cohort. The limitation was that data on medication changes were unavailable.

Further investigation proving the utility of circulating endotrophin as a risk marker and potentially as an actor in disease progression will be important for designing and monitoring intervention strategies to reduce fibrosis and consequent organ function loss. Furthermore, it will be interesting to determine whether changes in endotrophin can predict a clinically meaningful response to therapies with kidney and cardiovascular outcomes benefit.

In conclusion, we validated the previous findings of endotrophin in type 1 diabetes in a large and unselected cohort. Higher levels of serum endotrophin, released during collagen type VI formation, were independently associated with a higher risk of mortality and development or progression of CKD in persons with type 1 diabetes. Urinary endotrophin was not associated with development of the specified complications.

## Data Availability

The original contributions presented in the study are included in the article/Supplementary Materials, further inquiries can be directed to the corresponding author.
