# Supplementary material for: Endotrophin as a risk marker of mortality and kidney complications in a type 1 diabetes cohort
Source: Front Mol Biosci. 2023 Sep 1;10:1229579. doi: 10.3389/fmolb.2023.1229579 (PMC10505392; doi:10.3389/fmolb.2023.1229579)
Supplement: Supplementary file 1 [file Table1.DOCX]

Supplementary Material

Endotrophin as a risk marker of mortality and kidney complications in a type 1 diabetes cohort

**Alexandra Louise Møller^1,2*^, Ninna Hahn Tougaard^3^, Daniel Guldager Kring Rasmussen^1^, Federica Genovese^1^, Pernille Falberg Rønn^3^, Tine Willum Hansen^3^, Morten Asser Karsdal^1^ and Peter Rossing^3,4^**

^1^Nordic Bioscience, Herlev, Denmark, ^2^Department of Biomedical Sciences, Faculty of Health and Medical Sciences, University of Copenhagen, Copenhagen, Denmark, ^3^Steno Diabetes Center Copenhagen, Herlev, Denmark, ^4^Department of Clinical Medicine, Faculty of Health and Medical Sciences, University of Copenhagen, Copenhagen, Denmark

*** Correspondence:**Alexandra Louise Møller
[alm@nordicbio.com](mailto:alm@nordicbio.com)

**Supplementary Table 1.** Diagnosis and procedure codes included in the endpoints.

|  | **Diagnosis or procedure** | **Classifier** | **Codes** |
| --- | --- | --- | --- |
| **Kidney failure** | Kidney transplantation | ICD-8 | 99770  Y9509 |
|  |  | OPR | 57480 |
|  |  | ICD-10 | Z940* |
|  |  | NCSP | KKAS00  KKAS10 KKAS20 |
|  |  |  |  |
|  | Chronic dialysis | NCSP | BJFD2 |
|  |  |  |  |
|  | CKD 5 | ICD-10 | N185 |
|  |  |  |  |
|  | Kidney failure as cause of death | ICD-10 | N17-19 |
| **MACE** | Myocardial infarction | ICD-8 | 410-411 |
|  |  | ICD-10 | I21-24 |
|  |  |  |  |
|  | Coronary intervention | OPR | 30009  30019  30029  30039  30049  30059  30069  30079  30089  30099  30109  30119  30120  30129  30139  30149  30159  30169  30179  30189  30199  30200  30240  30241  30245  30280  30300  30350  30354  30359  30850 |
|  |  | NCSP | KFNA-KFNG (excluding KNFG20 +22) |
|  |  |  |  |
|  | Stroke | ICD-8 | 430-434, 436 |
|  |  | ICD-10 | I60-66 |
|  |  |  |  |
|  | Cardiovascular death | ICD-10 | I21-24  I60-66 |
| **Heart failure** |  | ICD-8 | 4270  78249 |
|  |  | ICD-10 | I50  I110  I130 |
| **Retinopathy** |  | ICD-8 | 24901  25001  376  37700-37703  37792  37878 |
|  |  | OPR | 16070  16080  16090  16100  16110  16510  16511  16512  16530  16540  16545  16580  16600 |
|  |  | ICD-10 | E103  E113  E133  E143  H33  H34  H36  H43 |
|  |  | NCSP | BCDE  BCHY8A  KCKC-KCKE  KCKW |

*OPR: Surgery and Treatment Classification. Used 1971-1995, ICD-8: International Statistical Classification of Diseases and Related Health Problems 8^th^ Ed. Used 1972- 1993, ICD-10: International Statistical Classification of Diseases and Related Health Problems 10^th^ Ed. Used from 1994, NCSP: Nordic Medico-Statistical Committee Classification of Surgical Procedures. Used from 1996. *Only included in prevalence, as diagnosis delay is possible.*

**Supplemental Table 2.** Clinical characteristics stratified by urinary endotrophin tertiles.

| **Characteristic** | **T1 (*n* = 417)** | **T2 (*n* = 417)** | **T3 (*n* = 417)** | ***P*** |  |
| --- | --- | --- | --- | --- | --- |
| Urinary endotrophin (ng/mmol) | 81 (58-98) | 171 (140-211) | 438 (317-672) |  |  |
| Age (years) | 54 ± 16 | 51 ± 15 | 52 ± 16 | 0.057 |  |
| Female sex (%) | 201 (48) | 217 (52) | 188 (45) | 0.132 |  |
| BMI (kg/m^2^) | 26 ± 3.7 | 25 ± 4.0 | 26 ± 4.3 | 0.172 |  |
| Systolic blood pressure (mmHg) | 130 ± 15.5 | 128 ± 15.0 | 131 ± 17.6 | 0.090 |  |
| Diastolic blood pressure (mmHg) | 76 ± 9.3 | 77 ± 8.5 | 76 ± 9.0 | 0.535 |  |
| Diabetes duration (years) | 27 ± 16 | 25 ± 15 | 28 ± 16 | **0.017** |  |
| HbA1c (mmol/mol) | 61.1 ± 11.6 | 61.9 ± 12.1 | 61.7 ± 12.6 | 0.587 |  |
| UAE (mg/g) | 5.5 (3.5-11.5) | 5.5 (3.5-10.5) | 5.5 (3.5-14.5) | 0.450 |  |
| eGFR (ml/min/1.73m^2^) | 91 ± 23 | 94 ± 22 | 92 ± 23 | 0.327 |  |
| LDL-cholesterol (mmol/L) | 2.4 ± 0.7 | 2.5 ± 0.7 | 2.4 ± 0.8 | 0.423 |  |
| Current smoker (%) | 67 (16) | 85 (21) | 61 (15) | 0.074 |  |
| **Treatment** | | | | |  |
| Insulin (%) | 412 (99) | 408 (98) | 411 (99) | 0.517 |  |
| Anti-hypertensives (%) | 199 (48) | 201 (48) | 222 (53) | 0.211 |  |
| RAAS blockade (%) | 170 (41) | 173 (42) | 205 (49) | **0.026** |  |
| Lipid-lowering medication (%) | 204 (49) | 210 (50) | 208 (50) | 0.914 |  |
| **Disease history** | | | | |  |
| MACE (%) | 50 (12) | 29 (7) | 58 (14) | **0.004** |  |
| Sight-threatening diabetic eye disease (%) | 90 (22) | 84 (20) | 89 (21) | 0.861 |  |

*Data are median (IQR), n (%), or mean ± SD. Urinary endotrophin levels are rounded to whole numbers due to sensitive personal data.*
